# Principles of stable isotope research – with special reference to protein metabolism

**DOI:** 10.1016/j.nutos.2021.02.005

**Published:** 2021-04

**Authors:** Daniel J. Wilkinson, Matthew S. Brook, Ken Smith

**Affiliations:** a MRC-Versus Arthritis Centre for Musculoskeletal Ageing Research, NIHR Nottingham BRC, UK; b Division of Health Sciences and Graduate Entry Medicine, School of Medicine, University of Nottingham, Royal Derby Hospital Centre, Derby, UK; c Division of Physiology, Pharmacology and Neuroscience, School of Life Sciences, Queen's Medical Centre, University of Nottingham, Nottingham, UK

**Keywords:** Muscle, Stable isotope tracers, Protein turnover, AA, Amino Acids, AP(E), Atom percent (excess), A-V, Arterial Venous, GC-MS, Gas Chromatography Mass Spectrometry, LC-MS, Liquid Chromatography Mass Spectrometry, MPS, Muscle Protein Synthesis, FBR, Fractional Breakdown Rate, FSR, Fractional Synthesis Rate, Ra, Rate of Appearance, Rd, Rate of Disappearance

## Abstract

The key to understanding the mechanisms regulating disease stems from the ability to accurately quantify the dynamic nature of the metabolism underlying the physiological and pathological changes occurring as a result of the disease. Stable isotope tracer technologies have been at the forefront of this for almost 80 years now, and through a combination of both intense theoretical and technological development over these decades, it is now possible to utilise stable isotope tracers to investigate the complexities of in vivo human metabolism from a whole body perspective, down to the regulation of sub-nanometer cellular components (i.e organelles, nucleotides and individual proteins). This review therefore aims to highlight; 1) the advances made in these stable isotope tracer approaches – with special reference given to their role in understanding the nutritional regulation of protein metabolism, 2) some considerations required for the appropriate application of these stable isotope techniques to study protein metabolism, 3) and finally how new stable isotopes approaches and instrument/technical developments will help to deliver greater clinical insight in the near future.

## 1 A brief history of stable isotope tracers – from deuterium and back again

**Fig. 1:**
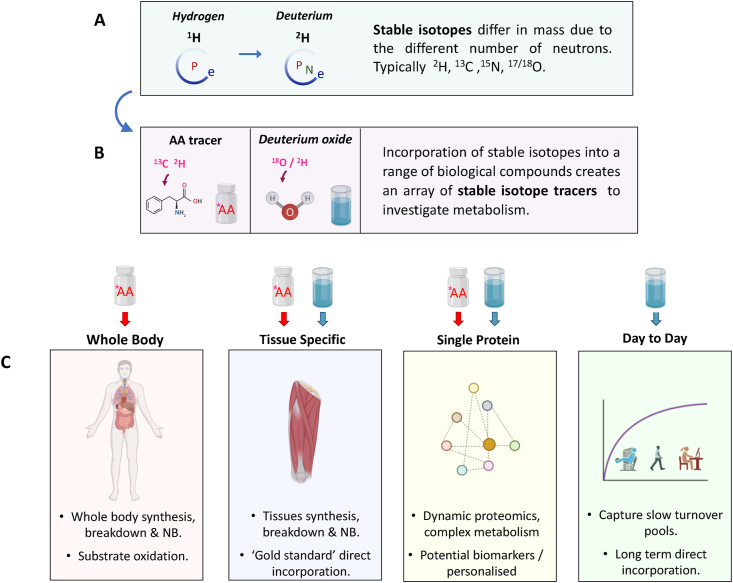
Summary schematic describing; A) what stable isotopes are, B) How they can be incorporated into biological tracers and C) the types and scale of mesurements that they can perform.

Stable isotopes have helped shape our understanding of human physiology, metabolism and disease for almost a century now, and it is the unique properties of stable isotopes that have enabled such progress. Stable isotopes are species of an element which whilst chemically and functionally identical, differ in mass due to the different number of neutrons in the atomic nucleus (Fig. 1). This difference in mass, measured using a technique called mass spectrometry, makes them analytically distinguishable from each other and allows them to be used to ‘trace’ metabolism. This feature of stable isotopes was quickly seized upon and provided a unique opportunity for two pioneering scientists; Rudolph Schoenheimer and David Rittenberg [1]. Following the discovery of the stable isotope of hydrogen – Deuterium, by a close colleague, Harold Urey [2], Schoenheimer and Rittenberg had the foresight to chemically exchange hydrogen positions on lipid molecules with its heavier stable isotope, deuterium, thereby making them ‘visible’ and identifiable to a mass spectrometer. By introducing “deuterated” linseed oil into the diet of mice, they were able to ‘trace’ how the mice absorbed and metabolised this lipid molecule *in situ* by tracking the appearance of deuterium labelled compounds into other metabolites [3]. Establishing the first use of what we now know as a ***stable isotope tracer.*** Using these tracer techniques, Schoenheimer and Rittenberg were able to rapidly (producing 14 papers on the topic in only a few years; [1]) uncover previously unknown facets involved in the regulation of mammalian metabolism; and by the end of the 1930s, with the identification and incorporation of the stable isotopes of carbon (^13^C; [4]), nitrogen (^15^N; [5,6]) and oxygen (^17^O and ^18^O; [7,8] into a range of biological compounds; there were an exquisite array of stable isotope tracer tools available to examine and understand the complexities of metabolism in health and disease [9].

Despite these exciting early developments, the expected expansion in the use of these metabolic tools was somewhat delayed by around 40 years, due in part to the discovery and utilisation of the easier, yet potentially more hazardous, radiolabelled isotopes and the lack of access to the appropriate analytical technology i.e. mass spectrometers, with the required sensitivity to analyse stable isotope tracers [10]. However, eventually, development of new, more sensitive mass spectrometry equipment, coupled with the hybridization to separation techniques, such as gas chromatography and liquid chromatography, finally enabled the widespread analyses of compounds from complex biological matrices [10]. Moreover, this specialist analytical equipment was also becoming more accessible to researchers outside of the pure physical chemistry fields they were initially designed for. Through the development of these new Gas Chromatography Mass Spectrometry (GC-MS) and Liquid Chromatography Mass Spectrometry (LC-MS) technologies, and the appreciation of the potential application of stable isotope tracer techniques, led by pioneers such as John Waterlow, Peter Garlick and Joe Millward in the late 60s and 70s, this helped to bring these methodologies back to the forefront of physiological and clinical research once more [11].

Extensive progress both technologically and methodologically has followed over the next half a century, thanks in large part to Waterlow *et al.* and many of their contemporaries; Dave Halliday [12], Mike Rennie [13], Bob Wolfe [14], Dennis Bier [15] and Dwight Mathews [16] to name only a few. As a result, we now know far more about the intricacies of metabolic regulation in both health and disease, and the impact effectors such as nutrition, exercise and hormones have on this. Moreover we now have an extensive array of stable isotope tracers available to probe a wide variety of metabolic processes, including substrate oxidation [17], synthesis of polymers [18]; including individual proteins/peptides [19] and nucleic acids [20], as well as now being able to image metabolism within a single cell structure [21]. Thereby, enabling robust and detailed investigation of most aspects of human metabolic function. It is doubtful that even Schoenheimer could have foreseen where his original experiments would have taken us to today. As such, a discussion of all facets of the application of stable isotopes in metabolic research is far beyond the scope of this review. Instead we will concentrate on their application to an area of physiological research where they have had significant impact; protein and amino acid metabolism. We will focus on the approaches and techniques used to determine whole body and skeletal muscle protein metabolism, including key factors to consider with regard to study design and ensuring that accurate/reproducible measurements are possible. Importantly, the approaches described in the next section of this review can be, with modification to the modelling kinetics, applied to the majority of substrate and metabolic flux measurements, i.e. lipid, sugar and nucleic acid metabolism, or indeed to the dynamic measurement of any metabolic pathway.

## 2 Technical application of stable isotopes to protein metabolic research; from whole body to single tissue

### 2.1 Whole body protein metabolism measures using stable isotope tracers

The initial renaissance in the use of stable isotope tracers in the study of the control of human metabolism from the 1960s and 70s grew from the interest in understanding the impact of protein deficiency and malnutrition in young children, a group in which the use of radiolabelled tracers was not possible or ethical [22]. As a result, methods were developed using the oral, intravenous or intragastric provision of ^15^N labelled amino acids (either ^15^N Gly, Ala or Lys; [12,23]) to measure whole body protein turnover via the excretion/flux of ^15^N labelled end products i.e. ^15^N enrichment in the ammonia and urea pools (the end products of amino acid metabolism); the flux (or dilution) of which reflects the whole body rate of protein breakdown [22,24]. Using knowledge of this, synthesis can then be derived from the balance of the processes diluting the tracer and removing the tracer from the amino acid pool using the following equations:

$$Equation\;1:\;Q\;=\left(\frac{\left(\frac{d}{Tr:T}\right)}{24\;\times \;BM}\right)$$

$$Equation\;2:\;PS=\;\left(\frac{Q-E}{24\;\times \;BM}\right)\times \;6.25$$

$$Equation\;3:\;PS=\;\left(\frac{Q-E}{24\;\times \;BM}\right)\times \;6.25$$

Equation4:NPB=PS−PB

(Q = flux, PS = protein synthesis, PB = protein breakdown, NPB = net protein balance, *d* = oral ^15^N dose (g glycine x 0.1972), Tr:T = tracer:tracee ratio, *E* = 24 h urinary nitrogen excretion, *I* = 24 h nitrogen intake provided by the controlled diet, BM = Body Mass, 6.25 = represents the conversion of nitrogen content to protein, average composition of N in amino acids).

Improvements in these initial applications have since been made and the primed constant infusion approach, using ^13^C labelled leucine or phenylalanine, both essential amino acids, has been adopted as the ‘gold standard’ [[25], [26], [27], [28]]. This approach involves priming the amino acid pool with a bolus of tracer thereby reducing the time needed to reach an isotopic steady state to 1–2 hours, permitting measurements over shorter periods (~2–3 hours), instead of the 24–30 hours needed previously using the ^15^N end product approaches. Thereby, enabling acute temporal measures of the effect of feeding (and any other short-term interventions) to be determined with relative ease. The benefits of whole body measures such as the ease of isostope administration, non/minimally invasive sampling and the potential for use under free living conditions (with appropriate caveats to ensure assumptions of the measures are met; [22]), make it ideally suited for study in the field of clinical nutrition and protein and amino acid metabolism. However there are a number of criteria in the design and implementation of these methods that need careful consideration.

The measurement of protein (or other substrate) oxidation, which is an important aspect in the measure of whole body protein metabolism, through release of ^13^CO_2_ from the ^13^C labelled AA, is an often overlooked measure, particularly when it comes to the effect of nutrition and disease. Overall the process is quite straightforward, yet it does require access to sensitive and specialist mass spectrometry instrumentation, such as an isotope ratio mass spectrometer. The ^13^C released from the AA into the breath as ^13^CO_2_ is diluted greatly by the oxidation of many other carbon based substrates such as glucose and fatty acids, meaning that the level of ^13^C tracer in the breath CO_2_ is very low ~0.005 atom percent excess (APE). It's also important to assess the impact of any given intervention on the fixation or release of carbon within the body and the contribution of the intervention, particularly nutritional, on the ^13^C labelling of the expired CO_2_. In the fasted state approximately 20–30% of the ^13^C label from the amino acid is retained (‘fixed’) in the body and not expired, however, during feeding less is retained [29]. Similarly some macronutrients e.g. beet vs cane sugar, have different ^13^C enrichment, so that failure to account for this, would lead to an erroneous estimate of ^13^C production [30], as such bicarbonate recovery studies should be performed under exactly the same conditions. In addition, it is normal to prime the bicarbonate pool, to ensure this pool also reaches a steady state quickly, thereby shortening the measurement period [31]. The measurement of oxidation also requires the measurement of the rate of CO_2_ production (VCO_2_) to calculate the total fractional oxidation, which can be achieved by means of indirect calorimetry and determination of breath CO_2_ production during the intervention. This is routinely accomplished using a metabolic monitor with a ventilated canopy or hood system, that is capable of measuring CO_2_, O_2_ and flow/volume [32]. Therefore, whilst whole body metabolism measures may seem straightforward and minimally invasive, analytical application and study design considerations can significantly impact interpretation, therefore understanding the intricacies of these measures is key for successful application.

### 2.2 Tissue/organ specific approaches to measure protein metabolism

While there are clear advantages for the whole body approach, particularly in the study of human nutrition health and disease, and patient groups, there are also significant limitations. For example, these measures act as a harmonic mean for the turnover of all tissues and organs of the body, and whilst certain larger organs such as skeletal muscle provide some of the greatest input to these measures, it is very difficult to distinguish which protein pools/tissues are driving those changes, nor how they may be differentially regulated by nutrition or other targeted interventions in health or disease. These limitations can be somewhat overcome through the introduction of tissue sampling (biopsy) from the tissue or organ of interest and/or arterial venous (A-V) sampling across the tissue bed (e.g. using femoral arterial(ised)/venous cannulation for the leg), to provide information on protein turnover, metabolism at the level of a tissue, organ or limb [[33], [34], [35]].

The A-V balance approach requires sampling of the arterial blood pool, as this is the source of blood/substrate delivery to all tissues and organs, and sampling of the venous drainage from that tissue, organ or limb, alongside a measure/estimation of the blood flow/perfusion to the tissue under investigation. At the simplest level, this approach through the determination of amino acid concentrations in arterial(-ised) and venous blood, using the Fick principle, and multiplying this difference by the blood flow to that tissue, will provide a measure of net balance (NB), indicating whether there is a net uptake (+ve balance) or net release (-ve balance) of amino acids across the organ/tissue or limb.

Equation5:NB=(Ca−Cv)×BF

(NB = net balance, Ca = concentration in the artery, Cv = concentration in the vein, BF = blood flow).

However this can be expanded upon through the addition of a stable isotopically labelled AA. With this, the rate of dilution of the tracer by the tracee (unlabelled amino acid) across the limb can be determined – this is simpler to achieve during a steady state approach - providing an estimate of the rate of appearance (Ra) of the AA, as a consequence of protein breakdown. Using the Ra and net balance measures, the rate of disappearance (Rd) of the tracer from the arterial pool or protein synthesis can be derived.

$$Equation\;6:\;\left(\left(\frac{Ea}{Ev}\right)-1\right)\;\times \;Ca\;\times \;BF$$

Equation7:NB=Rd−Ra

(Ra = rate of appearance, Ea = enrichment of tracer in the artery, Ev = enrichment of tracer in the vein, Rd = rate of disappearance).

Although there are many variations on how Ra and Rd are calculated; equations 5, 6 and 7 will provide the necessary outputs and in general they provide the same qualitative synthetic or proteolytic responses, ie. directionality of change, though not quantitatively i.e magnitude [36] as others approaches that have been proposed. It is important to consider the choice of AA to act as your tracer for A-V balance measures however, as it should not be subject to significant secondary metabolism within the tissue of interest. For example, phenylalanine is probably the tracer of choice, especially for measures of muscle protein turnover, given that phenylalanine metabolism is relatively simple in muscle, it is only incorporated into or released from protein and is not involved in intermediary metabolism (whereas its conversion to tyrosine by phenylalanine hydroxylase needs to be considered when using phenylalnine for whole body measures for example); leucine on the other hand undergoes reversible transamination to ketoisocaproate (KIC), and subsequent decarboxylation to CO_2_, so that the exchange of label has to be accounted for in KIC and CO_2_, greatly complicating the sampling and analysis.

In practice, the A-V balance approach works optimally under highly controlled conditions, i.e. a thermoneutral environment to prevent changes in blood flow/vessel dilatation, quiet and still, to prevent blood flow changes, alongside simultaneous arterial and venous sampling. Furthermore, if using the arm or leg, then the hand or foot should be occluded using a pressure cuff during blood flow measurement, to minimise contributions from other tissues and pools to both flow and metabolism. These highly controlled conditions can make this approach difficult to perform accurately, potentially leading to high variability in the data and the need for a multitude of assumptions for application within health and disease. Furthermore, with potential for contribution to the A or V pool from other tissues surrounding the organ of interest (skin, collagen etc.), the accuracy of the approach to reflect metabolism of muscle tissue only, may also be brought into question [36].

In contrast, the direct incorporation or “precursor-product” approach to measuring protein turnover is often considered the ‘gold standard’ for measuring tissue specific protein metabolic changes [12,13]. This technique requires the measurement of the amount of tracer incorporated into the “product” or tissue over time (usually sampled via surgical biopsy), and the labelling throughout this period in the pool from which the protein is made, the ‘precursor’. From this precursor – product relationship the fractional synthesis rate (FSR) can then be calculated using the following equation:

$$Equation\;8:\;FSR\left(\%.h^{-1}\right)=\left(\frac{Em2-Em1\;}{Ep}\;\times \frac{1}{t}\right)\times \;100$$

(Em2 = enrichment of tracer in muscle at timpoint 2, Em1 = enrichment of tracer in muscle at timepoint 1, Ep = average tracer enrichment in precusor pool across sampling period, t = time).

Ideally this “precursor” would be the labelling in the aminoacyl-tRNA pool, but this is technically difficult, but not impossible, due to the instability and small size of this pool [37]. Therefore, surrogates have been widely used, the keto-acid of leucine, KIC, is formed in muscle from leucine, released into venous plasma, and therefore is a reasonable estimate of intramuscular leucine labelling when using leucine based stable isotope tracers, and can be readily sampled many times during the study [38]. Phenylalanine is the most widely used amino acid to measure MPS, due to it’s limited metabolism within muscle, but as there is no intracellular derived surrogate like leucine, plasma labelling of phenylalanine is often times used as the precursor, yet this can significantly underestimate the absolute FSR [39,40]. As such, measuring the intracellular pool is the preferred approach, but this requires multiple biopsies and an isotopic steady state to minimise significant fluctuations in the precursor pool, making the experiment technically more challenging and more invasive than those with leucine tracers. Importantly, both approaches for leucine and phenyalanine surrogates, in our hands, give very similar measures of fasting and fed rates of MPS indicating any errors in estimating the precursor using these approaches are very small. Previous attempts to overcome the uncertainty around the measurement of the ‘true’ precursor for protein synthesis by flooding with a large dose (3g) of highly labelled amino acid (20 atom percent) to equalise the labelling in all the possible precursor pools [41,42] were unsuccessful due to the fact that the amino acid chosen as the flooding amino acid; phenylalanine or leucine, caused a significant stimulation of synthesis thereby negating the primary aim of a tracer, not to influence the metabolism of the pathway it was tracing. Subsequent studies demonstrated that other EAA also induced a stimulation of MPS [43,44] whereas NEAA did not e.g. Proline, Glycine [44,45] supporting the validity for the use of the flooding approach, with the appropriate choice of amino acid. Yet one needs to consider the potential for secondary metabolism of these NEAA in vivo also.

Finally, whilst many of the protein metabolic derangements in health and disease are proposed to be driven primarily through an impairment within the regulation of protein synthesis pathways [46,47]. A significant part of this conclusion is largely driven via the technical difficulties in accurately measuring tissue specific protein breakdown rates [48]. Through A-V balance, we can assess the Ra, however this measure is confounded by numerous assumptions, with one major one being that all AA being released into the venous circulation are derived from the breakdown of protein (i.e do not undergo recycling) from one tissue (i.e muscle) and not surrounding tissue beds (skin, collagen etc..). This was somewhat overcome however, by the development of the fractional breakdown rate measure by Wolfe and Chinkes in the 1990's to directly compliment the tissue specific FSR approach. Wolfe and Chinkes devised a way to measure FBR directly in the tissue using a mix of staged stable isotope tracer administrations of the same AA with differing isotopic labels (ie. ^13^C, ^15^N and D). By following their rate of decay (through dilution via the unlabelled AA [49,50] within the intracellular and plasma pools [49,50]), a FBR can be determined (please see [9] for more details of this measure and its derivation). This represented a major step forward in our understanding of tissue specific protein breakdown. However, this method remains technically challenging to perform, involves invasive sampling and the need for administration of large amounts of differently labelled tracers, therefore it remains rarely used compared to its FSR counterpart. Work on less costly and invasive measures of FBR are still therefore needed, and are beginning to now become available ([51]).

As a result of these stable isotope tracer techniques we now know that the tissue pools are continually under a constant state of turnover, with each tissue turning over at significantly differing rates dependent on their role in the body; for example, splanchnic tissues can turnover at rates of ~50%/d, while skeletal muscle turns over much slower at around 1–1.5%/d; [12,52], and is acutely regulated during periods of feeding and fasting [53]. Moreover, due to the important metabolic contribution of this organ, tracer-based studies involving skeletal muscle have dominated in recent years and regulation of its metabolism by nutrition, exercise, and hormones is now well understood (as summarised in Table 1 and described in other papers in this series) yet there is still much that remains to be uncovered. Moreover, the recent availability of multiply labelled ^13^C amino acids e.g. 1,2 ^13^C_2_ Leucine or U–^13^C_6_ phenylalanine, which increases the labelling of the protein/tissue of interest, has helped to enhance the temporal resolution of these approaches enabling measurement of tracer incorporation into slow turning over tissues such as muscle over as little as 45mins, such that we can now define the temporal response of muscle protein synthesis to protein/interventional feeding with far more accuracy. Therefore, with the diversity of different substrate based stable isotope tracer approaches currently available combined with the extensive methodological/analytical development already achieved, what does the future hold for stable isotope tracers in metabolic research?

**Table 1:** Summary table highlighting some of the important findings relating to our understanding of the control of in vivo protein metabolism that stable isotope approaches have help to uncover. This is not meant to be an exhaustive list of all studies within this area, and we apologise to those authors work which we have not been able to include here.

Reference	Stable isotope tracer approach	Major findings
***Rennie et al.,*** [13]	PCI – MPS & WBPT - L-[1–^13^C]-Leucine	Mixed meal doubles MPS = more than half total protein synthesis in whole body – First validation of approach
***Bennet et al.,*** [95]	PCI - MPS - L-[1–^13^C]-Leucine	IV AA stimulate MPS
***Smith et al.*,** [43,44]	PCI - MPS - L-[1–^13^C]-Leucine	Individual EAA, i.e. Leu but not NEAA stimulate MPS
***Battezzati et al.,*** [96]	IV and NG infusion of [1–^13^C] Alanine Whole body utilisation and splanchnic uptake	Little NEAA survive first pass extraction across splanchnic tissues and become available to peripheral tissues/organs
***Bohe et al.,*** [97]	PCI - MPS - [D_3_]-Ketoisocaproate	‘Muscle Full” phenomenon first shown – ivAA stimulation of MPS over 90–120 min, then returns to baseline/fasted MPS
***Cuthbertson et al.,*** [98]	PCI - MPS - L-[1–^13^C]-Ketoisocaproate	Muscle anabolic resistance of MPS to EAA in older muscle
***Greenhaff et al.,*** [99]	PCI - MPS - L-[1,2–^13^C_2_]-Leucine & AV Balance - [^2^H5]-Phenylalanine	Insulin does not stimulate MPS, main effect is inhibition of MPB.
***Atherton et al.,*** [100]	PCI - MPS - L-[1,2–^13^C_2_]-Leucine	‘Muscle full’ confirmed – oral Protein load – using IRMS for increased sensitivity
***Kumar et al.,*** [101]	PCI - MPS - L-[1,2–^13^C_2_]-Leucine	Muscle anabolic resistance of MPS to acute resistance exercise in older men
***Wilkinson et al.,*** [102]	PCI - MPS - L-[1,2–^13^C_2_]-Leucine and AV Balance using [^2^H_5_]-phenylalanine	Leucine and its metabolite HMB both maximally stimulate MPS, HMB suppresses MPB.
***Phillips et al.,*** [103,104]	PCI - MPS - L-[1,2–^13^C_2_]-Leucine and A-V Balance using [^2^H_5_]-phenylalanine	Improved AA delivery via RET or pharmacological approaches do not impact MPS responses
***Wilkinson et al.,*** [105]	Oral D_2_O bolus - iMPS	D_2_O provide similar rates of MPS to labelled AA approaches stimulation of MPS by RE detectable over 2 days
***Mitchell et al.,*** [106,107]	PCI - MPS - L-[^13^C_6_]-phenylalanine	Neither delivery profile of EAA (pulse v bolus), nor blood flow to the muscle impact MPS responses in Older muscle
***Wilkinson et al.,*** [74]	Oral D_2_O bolus - MPS, & PCI - MPS - L-[^13^C_6_]-phenylalanine	Increased D_2_O dosing permits acute measures of MPS, when coupled with IRMS - comparable to AA tracers.
***Brook et al.,*** [108]	Oral D_2_O bolus/weekly top-up – iMPS	D_2_O chronic measures reveal increased iMPS in response to RET occur early i.e. 0-3 weeks
***Brook et al.,*** [109]	Oral D_2_O bolus/weekly top-up – iMPS	D_2_O – multivalent tracer used to measure turnover of other polymers i.e.RNA – revealing deficits in iMPS and Ribosomal biogenesis in older muscle
***Brook et al.,*** [20]	Oral D_2_O bolus/weekly top-up – iMPS	Development of a novel approaches to quantify RNA turnover using D_2_O
***Wilkinson et al.,*** [110]	PCI - MPS - L-[^13^C_6_]-Phenylalanine	Leucine enriched (40%) EAA containing as little as 0.6g of leucine are sufficient to acutely stimulate MPS.
***Gharadaghi et al.,*** [111]	Oral D_2_O bolus/daily top-up – iMPS	Exogenous testosterone overcomes anabolic resistance to RET, increased iMPS, in older men.

PCI - Primed Constant Infusion, MPS - Muscle Protein Synthesis via direct incorporation, iMPS – integrated Muscle Protein synthesis. Could also be total mixed muscle or myofibrillar specific protein synthesis. WBPT - Whole Body Protein Turnover. AV balance – Breakdown, Net balance and Synthesis. RE/RET – Resistance Exercise/Training.

## 3 Methodological developments and future applications

As with many areas of science, progress is regulated by technological development. In the case of stable isotope tracers this relates, in the most part, to mass spectrometry, the primary route for analyses. The sensitivity, selectivity and accuracy of mass spectrometric instrumentation for the assessment of small shifts in isotopic abundance, has increased by several orders of magnitude over recent years. Furthermore with the introduction of instrumentation such as GC-combustion-isotope ratio mass spectrometry (IRMS) in the late 1980s [54], GC-pyrolysis-IRMS in the 1990s [55] and high resolution FT/Orbitrap mass analysers in the early 2000's [56], it is now possible to measure excess isotopic abundances down to the level of 0.0005APE increasing temporal resolution further. In addition, we now also have the analytical capability to separate the isotopologues of H, C and N from each other [57], and thereby measure different isotopes within the same molecule/fragment. This progress and improvement in sensitivity is particularly important when it comes to the measurement of slowly turning over metabolic pools such as skeletal muscle protein, bone collagen and DNA. Where incorporation of the stable isotope label will occur slowly and changes in measured isotopic abundance are extremely small as a result [20,58,59]. These developments have as such, greatly enhanced our abilities to understand the important yet complex facets of human skeletal muscle physiology, and how it plays an often overlooked yet highly important role in maintaining metabolic health across the life span [18,60,61]. Moreover these advancements have also helped to re-introduce a crucial stable isotope approach, one of the first to be developed during the initial early works of Schoenheimer and Rittenberg, yet the benefits of which have only just begun to truly be recognised in the last decades of the 20^th^ Century – Deuterium Oxide (D_2_O; [62]).

The benefits of D_2_O as a stable isotope tracer are clear to see. Orally administered - meaning there is no need for invasive intravenous infusions, under laboratory controlled conditions, thereby allowing wider application within populations where traditional substrate specific stable isotope tracers may be contraindicated or difficult to implement - it rapidly equilibrates throughout all the body water pools e.g. blood, intracellular water, saliva, any of which can be sampled as the surrogate precursor, and has a long half-life (~11 days). The deuterium from body water can then be incorporated/exchanged onto different substrates at stable C–H positions through biological reductions during de novo synthesis, and the metabolic flux of these substrate pools can then be determined from measurement of the amount of the label that is incorporated into the end product, whether it be glucose, lipid, DNA or protein [20,[63], [64], [65]]. Because D_2_O is not substrate specific i.e. it can label multiple substrates, and the body water turnover rate is relatively slow, the rate of turnover of multiple substrates can be monitored simultaneously over periods from a few hours (for fast turnover compounds like glucose), to days (for lipids) to weeks or months (for slow turnover compounds like proteins and DNA) within a single biological sample. As such, findings from D_2_O stable isotope tracer research has far more applicability to the real world, and the study of both healthy and individuals with disease in the free living state. Moreover, it shows great applicability for the study of integrated or cumulative, temporal protein metabolic responses to chronic clinical nutritional and/or pharmaceutical interventions, as highlighted in recent work from our labs and others [[66], [67], [68], [69]]. A vast amount of novel information has been generated as a result in the study of skeletal muscle protein turnover and the processes regulating it, in health and disease, using the D_2_O tracer approach over recent years [18,[70], [71], [72]]. It is also important to note, that these D_2_O based tracer approaches have been extensively validated and show good agreement with rates of fasted – fed MPS measured using traditional substrate specific AA tracers [73,74]. While cumulative rates per day, equate to the average of the fasted-fed cycle throughout the day, i.e. 1.4%/day; 0.045 %/h fasted plus 0.09%/h for ~2 hours post feed, equivalent to 0.06%/h over the day.

Muscle, as with other tissues, consists of thousands of individual proteins, all with a multitude of varying biological roles, and therefore their turnover will be vastly different. Therefore, when assessing experimental changes in skeletal muscle protein turnover in response to health, disease or clinical intervention, measuring changes within specific proteins rather than the bulk fraction in which they are contained, will undoubtedly better reflect the biology of the condition under scrutiny, leading to the identification of important and previously unknown metabolic networks which may be regulating this condition. In the situation of human disease this could help identify new biomarkers that would define new treatment paradigms. Indeed such novel approaches have already provided insight into previously unknown facets regulating human health such as cardiac remodeling [70], lipid dynamics to avoid pathogenesis of cardiovascular disease and non-alcoholic fatty liver disease (NAFLD; [72]), and potential biomarkers for Alzheimer's/Dementia [75,76]. Therefore, the capability of measuring the turnover rates of 1000's of individual proteins, so-called ‘dynamic proteomics’, within a sample is now an achievable and exciting area of focus for clinical researchers going forward [77].

Dynamic proteomics was initially developed using deuterium labelled amino acids provided to in vitro culture or ^15^N labelled algal cells within animal diets, which while ideally suited to pre-clinical models, application to humans in vivo was limited or difficult to achieve [10]. However, it wasn't long before the previously identified benefits of using D_2_O were recognized, greatly extending the capacity and coverage of this dynamic proteomics approach [71,77,78]. Regular dosing with D_2_O, would label endogenous non-essential AA and these ‘deuterated’ AA are then incorporated into peptides and proteins. With knowledge of the initial predicted precursor labelling (based on body water levels), and the peptide amino acid composition i.e. knowledge of the number of each theoretically labelled AA, rates of turnover of individual peptides and hence the parent proteins, can be calculated [77,79]. This has been extensively validated in animal and cell culture models [[80], [81], [82]] and more recently in humans too [71,78,83]. As a result these techniques are now being used to measure skeletal muscle proteome dynamics across a wide range of conditions enabling better understanding of specific changes in skeletal muscle protein metabolism in response to exercise [[83], [84], [85]], the impact of caloric restriction and ageing on skeletal muscle mitochondrial proteome dynamics [69,86], and the effects of pharmacological interventions on the skeletal muscle proteome [69,87], enabling a deeper understanding of the complex regulation of protein metabolism in health and disease [18]. Although these measurements remain challenging, a key benefit of these approaches will clearly lie in their potential for providing individualised information towards a greater degree of personalised medicine. We know that humans are incredibly heterogeneous in their physiological responses, responding in vastly different ways to the onset of some diseases, applications of therapies and provision of nutrition [[88], [89], [90], [91]]. Understanding in greater detail what underlying processes are driving these individualistic responses, through the use of innovative stable isotope approaches and analytical techniques such as dynamic proteomics will greatly assist researchers and clinicians in personalising the most appropriate and effective course of treatment, with the aim of improving patient outcomes. Moreover, with such detailed and in depth insight into the underlying mechanisms provided by dynamic proteomics, it is possible that previously unknown therapeutic targets may also be identified. The potential of this novel and exciting application for stable isotope tracers is clear, and it will no doubt become an incredibly powerful and useful approach in the near future. Although access to the technology and facilities to perform such work is currently restricted to a few specialist labs. The development of new high resolutions MS equipment continues apace, and with individual researchers making the complex computational steps for data analyses of dynamic proteomics data into open access, user friendly software packages; such as ProTurn [92], D2Ome [93] and Deuterater [94]. The potential for researchers to implement these important innovative technologies within research going forward is, and will continue to become, more possible, providing an exciting and rich future within the field of stable isotope tracer based clinical research. As exemplified in the following collection of articles.
