# Erratum to “Principles of Stable Isotope Research – with Special Reference to Protein Metabolism” [Clinical Nutrition Open Science 36C (2021) 111–125]

**DOI:** 10.1016/j.nutos.2022.04.001

**Published:** 2022-10

**Authors:** Daniel J. Wilkinson, Matthew S. Brook, Ken Smith

**Affiliations:** a MRC-Versus Arthritis Centre for Musculoskeletal Ageing Research, NIHR Nottingham BRC, UK; b Division of Health Sciences and Graduate Entry Medicine, School of Medicine, University of Nottingham, Royal Derby Hospital Centre, Derby, UK; c Division of Physiology, Pharmacology and Neuroscience, School of Life Sciences, Queen's Medical Centre, University of Nottingham, Nottingham, UK

The publisher regrets the Conflict of Interest statement was not included in the published version of this article.

The authors declare no conflicts of interest.

The publisher would like to apologise for any inconvenience caused.

